# Cockles, barnacles and ascidians compose a subtidal facilitation cascade with multiple hierarchical levels of foundation species

**DOI:** 10.1038/s41598-017-00260-2

**Published:** 2017-03-22

**Authors:** Eugeniy Yakovis, Anna Artemieva

**Affiliations:** Invertebrate Zoology Department, St.-Petersburg State University, 199034 St.-Petersburg, Russia

## Abstract

Facilitation cascades occur when multiple foundation species in a community are involved in a hierarchy of positive interactions, and consist of a primary facilitator which positively affects secondary facilitators, each supporting a suit of dependent species. There is no theoretical limit to the number of levels in a facilitation cascade, yet the existence of more than two has rarely been examined. We manipulated biogenic substrate produced by a primary facilitator (cockle shells) and a secondary facilitator (barnacles and their empty tests) in a space-limited subtidal community to test the hypothesis that solitary ascidians would be the third-level facilitator. In the field, most ascidians were found on barnacles, and most barnacles occupied cockle shells. To produce this pattern, barnacles could nurse ascidians (a longer ‘facilitation chain’) or outcompete them from cockle shells (a shorter chain). Experimental results clearly supported the nursing hypothesis providing evidence for a facilitation cascade with three hierarchical levels of foundation species. Our findings confirm that like predation and competition, positive interspecific interactions nest into multi-tier hierarchies with numerous levels. While the number of foundation species should increase community stability and resilience as it increases diversity and reduces environmental stress, facilitation chain length may have the opposite effect.

## Introduction

Interspecific interactions have long been recognised to generate multi-level hierarchies or complex webs of species in natural communities. These models, however, have mainly been applied to negative interactions: predator-prey relationships that constitute food chains or webs^1^, and competitors that develop competitive hierarchies or networks^2, 3^. Only recently have empirical studies revealed the critical role of positive species interactions in community organisation^4^. Growing interest in mutualistic networks^5^, foundation species^6, 7^ and ecosystem engineering^8^ has helped uncover how common and widespread communities are that are shaped by facilitators. In most of these communities foundation species modify or create habitat, providing conditions favorable for associated organisms. Initially focused on the systems with a single foundation species (e.g. mussels) or a functionally similar guild (e.g. corals, kelp or trees), this research has shifted to the communities shaped by multiple facilitators each interacting with others and supporting its own assemblage of dependent species^9–11^. An important conceptual consequence of this work is that positive interspecific interactions, like predation and competition, have been shown to potentially be multi-level hierarchical structures.

The term ‘facilitation cascade’ was introduced by Altieri *et al*.^9^ who explored New England cobble beaches, where cordgrass positively affects ribbed mussels, and each of them facilitates a suit of dependent species. In this system cordgrass is a ‘primary’ and mussels a ‘secondary facilitator’, and the authors suggested that many more facilitation cascades should exist. Naturally, several communities with cascading positive interactions from epibenthic^12–19^, infaunal^20^ and terrestrial^21, 22^ habitats have been studied since, confirming the generality of the phenomenon^23^. The hierarchies of positive interspecific interactions mediated by biogenic habitat provision are specifically named ‘habitat cascades’^13^.

With a single exception^19^, known facilitation cascades are comprised of only two hierarchical levels, primary and secondary, of foundation species, and the corresponing suits of numerous dependent species. Food chains, in contrast, commonly have up to 5–6 levels, their number being constrained by resource availability, type of predator-prey interactions, community history, ecosystem size, and disturbance level^24^. The only long ‘facilitation chain’ examined to date is a six-level intertidal habitat cascade developed by a bivalve, a green seaweed, a trochid snail, a bryozoan, and a red seaweed in the Avon-Heathcote Estuary, New Zealand^19^. Given the limited number of cascades studied, it is uncertain, if hierarchical positive interactions are fundamentally more restricted in number of levels than negative ones (e.g. by instability of longer chains), or most multilevel facilitation cascades have simply not been investigated. There is also a possibility that the relationships between secondary facilitators in some of the known cascades have been overlooked. Here we address this question with a field experiment to test the hypothesis that a subtidal epibenthic community is driven by the facilitation cascade with at least three hierarchical levels of foundation species, each providing habitat for numerous dependent species. Since the very presence of a facilitation cascade increases habitat complexity, species richness and abundance in a community^9, 10, 14, 21, 25^, we assume that the number of levels in a cascade combined with the number of coexisting functionally different foundation species would be powerful predictors of communtity structure and function.

Biogenic substrate often provides living space for sessile organisms on soft bottoms^26, 27^. In subtidal habitats, where natural disturbance is low, this can produce complex networks of interspecific biotic interactions mediated by space provision and utilisation. In the shallow subtidal of the White Sea (a semi-enclosed southern inlet of the Barents Sea in NW Russia) near Solovetsky Islands most biomass and diversity of macrobenthic organisms is concentrated on epibenthic patches of small hard substrate scattered over soft sediment^28^. These ‘primary’ substrates are either mollusk shells or pebble-like ice rafted dropstones, but the epibiota chiefly resides on secondary space providers, namely the co-dominant barnacles *Balanus crenatus* (live individuals and empty tests), solitary ascidians (*Styela* spp., *Molgula* spp. and *Boltenia echinata*), and several species of foliose red algae^10^. Barnacles, ascidians and algae act as foundation species each supporting a specific diverse nested assemblage of associated sessile organisms. As indicated by the number of growth rings on barnacle tests, individual epibenthic patches can persist at least for 9–10 years^29^. In 2001–2005 the Greenland cockle *Serripes groenlandicus* was a top supplier of primary substrates^10^. These bivalves are completely buried below the sediment surface while alive^30^, and their empty valves provide space for epibiota only once they die. Consequently, *Serripes* can facilitate sessile organisms far beyond the lifespan of an individual cockle, like reef-building corals and trees turning into nurse logs.

According to previous findings, the surface of primary substrate is overwhelmingly dominated by barnacles and their empty tests, which are also often attached to each other and form multi-tier clusters. Most ascidians reside on barnacles, empty barnacle tests and other ascidians. The largest individuals are mainly found on empty barnacle tests, while on primary substrates ascidians are scarce. Red algae grow both on ascidians and barnacles^10^. Given the proportion of the epibenthic patches based on empty *Serripes* shells, the primary hard substrates on unstructured seafloor, all the sessile organisms associated with these patches are facilitated by *Serripes* (directly or indirectly). The interactions between barnacles and ascidians, however, remain unclear^29^, and the patterns of space provision and utilisation have only been quantified for *Serripes* and barnacles^10^. We hypothesized that *Serripes*, barnacles, ascidians, and their associated assemblages of dependent species might comprise a facilitation cascade with at least three nested levels of foundation species.

To test this hypothesis we sampled three subtidal sites to confirm *Serripes* as a principal primary hard substrate supplier, analysed the field distribution of ascidian recruits and adults by microhabitat, and evaluated the relative contributions of different foundation species to substrate pool for each other and for all the dependent sessile organisms, to quantify the space-mediated interspecific relationships in the system. We then manipulated initially defaunated *Serripes* shells, live barnacles and barnacle empty tests to confirm or refute the facilitation of ascidians by barnacles, and thus assess the number of nested levels in the facilitation cascade. We assumed that the space utilisation pattern observed with barnacles dominating cockle shells and ascidians chiefly growing on barnacles could result from different processes. First, barnacles could outcompete ascidians on suitable or favorable primary substrates. In this case we expected similar or higher ascidian per area recruitment rates on cockle shells (initially empty or cleared of barnacles) than on barnacles. Second, barnacles could facilitate (nurse) ascidians otherwise incapable of autonomous colonisation of clear primary substrates. This would be manifested as higher abundance of ascidian recruits on the surface of barnacle tests regardless of the excess of empty cockle shells nearby. Evidence for facilitation would prove that ascidians are the third-order foundation species in the facilitation cascade with cockles and barnacles.

## Results

### Field sampling

In total, within the epibenthic patches found we recorded 132 species of sessile macrobenthic organisms (including 64 bryozoans, 18 algae, 18 coelenterates, and 11 ascidians), with 96 ± 1% of occurrences on 6 principal substrates: primary (20 ± 5%), live barnacles (39 ± 13%), their empty tests (8 ± 4%), solitary ascidians (17 ± 9%), red algae (10 ± 5%) and discord mussels (1 ± 0%). Primary substrates were mostly of biogenic origin. Per 1 m^2^ of the bottom, *Serripes groenlandicus* empty shells contributed 0.026 ± 0.011 m^2^ (66 ± 8%) of primary substrate area and other mollusk shells provided 0.004 ± 0.002 m^2^ (10 ± 3%) more, while stones accounted for 0.007 ± 0.002 m^2^ (22 ± 11%). *Serripes* shells constantly prevailed as primary substrates across the locations, on average contributing 80 ± 2%, 67 ± 8% and 52 ± 2% of primary substrate area at Sites 1, 2 and 3, correspondingly. The proportion of primary substrate contributed by *Serripes* at Site 1 had almost no correlation with sampling date in 2001–2014 (*R* ^*2*^ = 0.016). *Balanus crenatus* numerically dominated on primary substrates. A typical epibenthic patch was a multi-tier cluster, in which barnacles and their empty tests directly occupied a cockle shell partially embedded in muddy sand convex side up, as other sessile organisms resided in upper tiers (Supplementary Fig. S1). Either live barnacles or their empty tests were present in 90 ± 5% (95 ± 1%, 80 ± 3% and 93 ± 2% at Sites 1, 2 and 3, resp.) of the epibenthic patches found on the bottom. 22 ± 6% (24 ± 2%, 11 ± 5% and 32 ± 1% at Sites 1, 2 and 3, resp.) of the epibenthic patches were fragments without a primary substrate, mostly based on wrecked empty barnacle tests.

**Figure 1:**
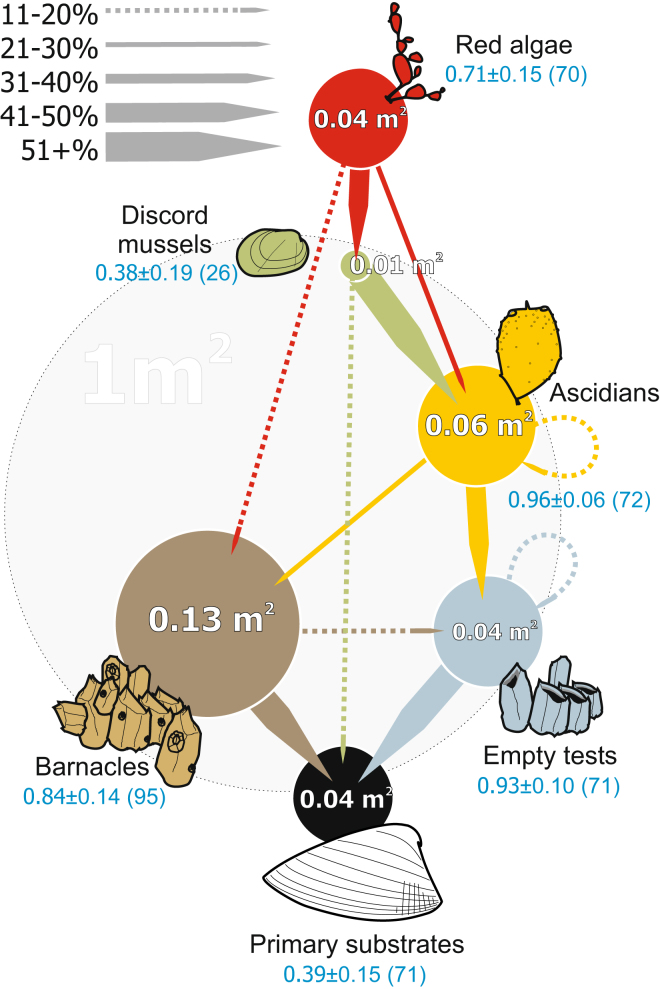
Substrate surface area provision and utilisation by foundation species (FS) in the field. Primary substrates: mollusk shells and pebbles, chiefly empty *Serripes groenlandicus* shells. Barnacles: *Balanus* spp., chiefly *B*. *crenatus*. Empty tests: chiefly left by *B*. *crenatus*. Ascidians: solitary ascidians *Molgula* spp., *Styela* spp., *Dendrodoa grossularia*, *Halocynthia pyriformis*. Discord mussels: *Musculus discors* and their nests. Red algae: *Phycodrys rubens, Euthora cristata, Odonthalia dentata, Cocottilus truncatus, Ptilota gunneri, Fimbrifolium dichotomum, Polysiphonia stricta*. Circle areas denote the mean approximate substrate area (m^2^) provided by the corresponding FS to other sessile species per m^2^ of the bottom. Arrow widths denote the mean fraction of this area provided by individuals that occupy the underlying substrate the arrow points to. Blue numbers: Shannon log-e diversity of the depended species associated with the FS ± S.E. In parentheses: the total number of sessile species found on the FS. All the mean values are averaged for the 3 sites, within-site variation pooled, except the arrow widths for discord mussels based on the average for sites 1 and 3 (no by-substrate measurements available for the Site 2). A grey 1 m^2^ circle denotes the scale.

Contributions of different foundation species to the substrate pool for each other and for the rest of sessile organisms, and the diversity of associated epibenthic assemblages are summarised in Fig. 1. While primary substrate offered 0.04 ± 0.01 m^2^ of surface per m^2^ of the muddy bottom (with 0.061 ± 0.004, 0.025 ± 0.006 and 0.027 ± 0.004 m^2^ at Sites 1, 2 and 3, resp.), the secondary contributors provided 3–9 times more (0.51 ± 0.05, 0.07 ± 0.01 and 0.24 ± 0.00 m^2^ at Sites 1, 2 and 3, resp.). Per m^2^ of the bottom, 72 ± 7% of estimated substrate surface area of the total 0.13 ± 0.07 m^2^ provided by live barnacles and 72 ± 8% of the total 0.04 ± 0.02 m^2^ supplied by their empty tests were contributed by the individuals attached to primary substrates. Solitary ascidians provided approximately 0.06 ± 0.02 m^2^ · m^−2^ of substrate, which was, in contrast, chiefly contributed by the individuals attached to empty barnacle tests (48 ± 4%) and live barnacles (26 ± 12%). Other contributors of substrate surface were the nests of discord mussels, commonly embedded in ascidian tunics, and the red algae growing on mussel nests and ascidians. The assemblages directly associated with primary substrate and discord mussels were much less diverse (*H*’ < 0.40) than those supported by barnacles, their empty tests and ascidians (*H*’ > 0.80).

In terms of biomass, most ascidians were attached to empty barnacle tests with a mean weight of 31 ± 14 g per m^2^ of the bottom and 738 ± 201 g per m^2^ of empty tests’ surface (Supplementary Fig. S2). Barnacles and their empty tests as a substrate together yielded 73 ± 11% of the total ascidian biomass. Species composition of solitary ascidians was similar on primary substrates, live barnacles and their empty tests where the dominating *Styela* spp. (cheifly *S*. *rustica*) contributed 80 ± 10%, 77 ± 5% and 69 ± 17% of the total ascidian biomass, correspondingly. *Molgula* spp. (cheifly *M*. *retortiformis*) was less abundant there with 14 ± 9%, 12 ± 7% and 27 ± 15%. Ascidians growing on other ascidians were, in contrast, dominated by *Molgula* spp. (53 ± 21%) followed by *Styela* spp. (31 ± 15%), and on red algae there was 41 ± 8% of *Molgula* spp., 32 ± 6% of *Boltenia echinata*, and only 10 ± 5% of *Styela* spp. On *Musculus* nests *Styela* spp. dominated with 55 ± 23%, followed by *Boltenia echinata* (29% ± 24%) and *Molgula* spp. (16 ± 16%).

Small ascidians, weighing 0.001 g and less, mostly occurred on live barnacles (Supplementary Fig. S3) with a mean abundance of 167 ± 112 ind.·m^−2^ of the bottom, which was 22 ± 9 m^−2^ or less for any other substrate. 67 ± 1% of ascidian recruits on live barnacles were *Styela* spp. Out of the small ascidians found on live barnacles, 32 ± 3% (data pooled by sample, n = 6) on Site 1 and 31 ± 2% (data pooled by sample, n = 2) on Site 3 were located inside an orifice. The other 45 ± 5% (Site 1) and 48 ± 2% (Site 3) were attached inside the folds on outer surface of immobile plates, and the rest were located openly on their outer surface.

The density of small ascidians per substrate surface area was extremely variable between the sites and relatively high on live barnacles compared to that on primary substrates, empty barnacle test, ascidians and discord mussels (Supplementary Fig. S3). In addition, the density of small ascidians (cheifly *Molgula* spp. and *Boltenia echinata*, with only 12 ± 4% fraction of *Styela* spp.) was exceptionally high and variable on red algae blades (where the abundance of adults was almost negligible, see Supplementary Fig. S2). Per substrate surface area, the number of small ascidians was significantly higher on live barnacles than on primary substrates at Site 1 (383 ± 12 vs 101 ± 6 m^−2^, p = 0.000008, Wilcoxon matched pairs test, Z = 4.46, n = 28) and Site 2 (260 ± 37 vs 69 ± 19 m^−2^, p = 0.043, Wilcoxon matched pairs test, Z = 2.02, n = 6). At Site 3 their number on live barnacles was also higher, (1345 ± 14 vs 567 ± 343 m^−2^), but the sample size was too small (n = 2) for a meaningful statistical comparison.

### Substrate manipulation experiments

#### Effect of substrate on ascidian recruitment rate

**Figure 2:**
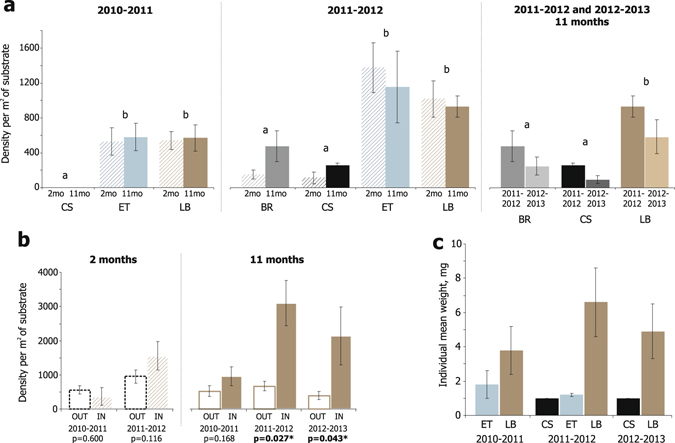
Recruitment of solitary ascidians by substrate type in the substrate manipulation experiment. Mean numbers of ascidian recruits on cockle shells with barnacles removed (BR), empty cockle shells (CS), empty barnacle tests (ET), and live barnacles (LB) with post-hoc test results indicated by lettering (**a**) (analyses of the data given in Table 1). Mean numbers of ascidian recruits on live barnacles inside (IN) and outside (OUT) the orifice per 1 m^2^ of substrate surface by year and exposure term pairwise compared by Wilcoxon matched pairs test (**b**). Mean individual weights of ascidian recruits on cockle shells (CS), empty barnacle tests (ET), and live barnacles (LB), exposed for 11 months (**c**).

In the substrate manipulation experiment, the density of ascidian recruits was similarly high on empty barnacle tests in ET-treatments and live barnacles in LB-treatments, and several times lower on empty cockle shells in CS- and BR-treatments regardless of the exposure (Fig. 2a, Table 1). The Treatment effect was the only significant one both in the first and the second year of the manipulations. Particularly, in 2010–2011, when the overall recruitment rate was lower, there were no ascidians in CS, while in 2011–2012 there were few in CS and BR. Ascidians were significantly more abundant in LB and ET in both respective years with no statistical difference between LB and ET and between the exposure terms. In the analysis of BR, ET and LB from the long trials of 2011–2012 and 2012–2013, the density of ascidian recruits was likewise significantly higher on live barnacles than on empty cockle shells and cockle shells with traces of barnacles (Table 1).

**Table 1:** Field substrate manipulation experiment: the results of ANOVA on abundances of solitary ascidians found on different substrates exposed for 2 and 11 months in 2010–2012, and for 11 months in 2012–2013.

Source of variation	df	SS	F	p	Mean ± S.E. by **Tr**eatment level (m^−2^)			
					BR	CS	ET	LB
2010–2011 (variances homogeneous, no transformation needed)								
**Tr**eatment (fixed)	2	2449056	7.00	**0.002**	—	0 ± 0 a	562 ± 115 b	558 ± 104 b
**Exp**osure (fixed)	1	7307	0.04	0.839				
**Tr** x **Exp** (fixed)	2	4232	0.01	0.988				
Error	42	7349452						
2011–2012 (variances homogeneous after square root transformation)								
**Tr**eatment (fixed)	3	3534	12.61	**0.000013**	309 ± 105 a	186 ± 43 a	1268 ± 240 b	976 ± 116 b
**Exp**osure (fixed)	1	79	0.85	0.364				
**Tr** x **Exp** (fixed)	2	299	1.07	0.377				
Error	32	2989						
2011–2012 (11 months only) and 2012–2013 (variances homogeneous after square root transformation)								
**Tr**eatment (fixed)	2	1380	7.49	**0.002**	328 ± 104 a	143 ± 38 a	—	758 ± 120 b
**Y**ear (fixed)	1	655	7.10	**0.013**				
**Tr** x **Y** (fixed)	2	6	0.03	0.967				
Error	28	2581						

Densities per m^2^ of substrate surface as a response variable. BR – *Serripes* shells with barnacles removed. CS – empty *Serripes* shells. ET – *Serripes* shells with empty barnacle tests. LB – *Serripes* shells with live barnacles. Ascidians found on *Serripes* shells in ET and LB treatments and on empty barnacle tests in LB treatments ignored. There were no ascidians on live barnacles in ET treatments. The results of Tukey HSD post-hoc tests are indicated by letters ‘a’ and ‘b’ following the means. Significantly (p < 0.05) different means have no letter in common.

There was no significant difference in ascidian density on cockle shell surface from bare shell (CS) treatments (94 ± 30 m^−2^) and shells with live barnacles (LB) treatments (141 ± 56 m^−2^) for the substrates exposed for 11 months in 2010–2013 (p = 0.674, F = 0.179) regardless of the year (Supplementary Table S1). The density of ascidians inside the orifices of the live barnacles exposed for 11 months in all LB from 2010–2013 (1787 ± 340 m^−2^) was significantly higher than their density on the outer surfaces of live barnacles (525 ± 90 m^−2^) (p = 0.0009, Wilcoxon matched pairs test, Z = 3.33, n = 24, three years pooled). Within the years, this difference was significant in 2011–2012 and 2012–2013, and nonsignificant in 2010–2011. It was also not significant for the 2 months exposure term regardless of the year (Fig. 2b). Out of the 327 ascidians totally found on live barnacles exposed for 11 months, 33% were located inside an orifice, 50% inside the folds on the outer surface of immobile plates, and the remainig 17% on their outer surface openly.

#### Species composition of ascidian recruits

The ascidian recruits in short-term manipulations were generally too small for accurate taxonomic identification. Out of the 507 ascidian individuals recorded in all the experimental units exposed for 11 months, there were 81% of *Styela*-like and 13% of *Molgula-*like recruits (the rest were *Boltenia echinata*). Although the proportion of *Molgula-*like recruits looked higher on cockle shell surface (totally 4 of 7 individuals in CS-treatments and 2 of 7 in LB-treatments), this microhabitat yielded too few ascidians for any statistical analysis. Ascidian species composition was similar on live barnacles and their empty tests (9% and 6% of *Molgula-*like recruits, respectively, p = 0.241, Chi-square, the data for 2010–2012 pooled, n = 420). The overall proportion shifted towards *Molgula* in 2012–2013, when we only exposed CS and LB units (37% on live barnacles, n = 70).

#### Effect of substrate on individual mean weight of ascidian recruits

The ascidian recruits were found on principal substrates of the three unit types (CS, ET and LB) only in 2011–2012. In 2010–2011 there were no ascidians in CS, and in 2012–2013 there was no ET-treatment. In 2011–2012, after 11 months of exposure, individual mean weight of ascidian recruits (averaged by unit) was similarly low on cockle shells in CS (0.0010 ± 0.0000 g, n = 4 units) and empty barnacle tests in ET (0.0012 ± 0.0001 g, n = 6 units) and significantly higher (0.0066 ± 0.0020 g, n = 6 units) on live barnacles in LB (Fig. 2c, Supplementary Table S2). Consistently, in 2010–2011 ascidians were also larger on live barnacles (0.0038 ± 0.0014 g, n = 10 units) than on empty tests (0.0018 ± 0.0008 g, n = 10 units; p = 0.029, Mann-Whitney U-test), and in 2012–2013 they were larger on live barnacles (0.0049 ± 0.0016 g, n = 5 units) than on cockle shells (0.0010 ± 0.0000 g, n = 3 units; p = 0.036, Mann-Whitney U-test) (Fig. 2c).

#### Effect of substrate on barnacle recruitment rate

There was no clear pattern in the variaton of barnacle recruitment rate by substrate, yet the abundance of juvenile barnacles experienced strong interannual fluctuations. Particularly, the density of barnacle recruits was highest on cockle shells in 2012–2013 (81489 ± 9701 m^−2^), when it was also substantial but significantly lower on live barnacles (21610 ± 4452 m^−2^; p = 0.001, Mann-Whitney U-test). There was no significant difference between any of the substrates in 2011–2012 (960 ± 702 m^−2^ on cockle shells, 844 ± 190 m^−2^ on empty tests, and 1417 ± 340 m^−2^ on live barnacles). In 2010–2011, the density of barnacle recruits on live barnacles (761 ± 147 m^−2^) significantly exceeded the one on cockle shells (138 ± 95 m^−2^), and was intermediate on empty tests (405 ± 114 m^−2^). The effect of exposure was nonsignificant both in 2010–2011 and 2011–2012 (Supplementary Table S3).

## Discussion

Consistent with previous observations^10^ our survey shows that the epibenthic community studied is structured by a space-mediated facilitation cascade with a primary (*Serripes groenlandicus*) and secondary (barnacles, solitary ascidians, discord mussels and foliouse red algae) foundation species. Our experiments clearly prove that barnacles, being the primary space holder, facilitate the recruitment of solitary ascidians, and the facilitation cascade consequently has at least three nested levels of foundation species.

Across the locations and years empty shells of *Serripes groenlandicus* constantly provided most primary hard substrate in terms of surface area, making one the primary foundation species in the macrobenthic community studied. Shells of live or dead invertebrates commonly serve as an essential source of living space for benthic sessile organisms in modern and ancient soft-sediment environments, creating the islands of increased abundance and diversity on the otherwise homogeneous seafloor^26, 27, 31^. In contrast to another known bivalve (*Austrovenus*) which also hosts a multi-level cascade in soft bottoms as a supplier of hard substrate for secondary sessile facilitators^19^, a live *Serripes* stays below the sediment surface, and its shell is only colonised post mortem. Like many other autogenic ecosystem engineers^8^, e.g. forest trees shedding their branches and trunks into streams or turning into nurse logs, *Serripes* modifies a habitat by producing physical structures that can last much longer than an individual cockle.

The highest diversity of attached epibiota was, however, directly associated not with primary, but with secondary biogenic substrates: barnacles, their empty tests and solitary ascidians (Fig. 1). Barnacles were the dominant primary space holders, and their presence alone more than four times increased the overall local availability of solid substrate, while ascidians, foliose algae and discord mussel nests added a comparable amount of flexible surfaces. Except for discord mussels, which are often deeply embedded into ascidian tunic, they all provide much more substrate space than occupy. Combined with the diversity of attached epibiota they sustain and the previously quantified difference between the assemblages they host^10^, this identifies them as secondary foundation species in the space-limited system. Given that both solitary ascidians and foliose algae are represented by several species, the facilitation cascade we explore shows the outstanding diversity of secondary foundation species compared to most other cascades studied to date, of which the most complex one (hosted by *Austrovenus*) has four^19^. While in many known cascades an effect of the primary facilitator at least partially involves amelioration of environmental stress like shading or reducing wave action^9, 14, 21^, similarly to *Austrovenus*, *Serripes* here clearly appears just a provider of living space.

The position of a primary space holder in space-limited systems is mostly occupied by the strongest competitor and is thusdetermined by negative interspecific interactions^32, 33^. The epibenthic species outcompeted from primary substrate may then retreat to the secondary one provided by a primary space holder itself^34^. In case some primary substrate is released from this dominant competitor by physical disturbance^32^ or keystone predation^35^, the weaker competitors temporally take over the vacant patches. The spatial structure we observed in the field (with barnacles monopolising primary substrates and other foundation species mainly occupying secondary ones) strongly resembled the typical pattern produced by competitive exclusion. Nonetheless, the distribution of ascidian recruits in the field samples and the outcome of the manipulations clearly showed the opposite ‘nursing’ hypothesis, i.e. the facilitation of ascidians by barnacles.

Our experiments evidence that live adult barnacles and their empty tests, both initially clear and with barnacles removed, similarly boost ascidian recruitment compared to bare *Serripes* shells. The recruitment of barnacles themselves, on the contrary, was not clearly affected by substrate type, and the defaunated primary substrates were intensively colonised by barnacles at least in 2011–2012 and 2012–2013. In addition, ascidians were larger on live barnacles than on their empty tests (and likely also than on *Serripes* shells, though the latter yielded too few ascidian recruits to know for sure). Nursing of ascidians by barnacles must hence involve several processes, one of them causing differential settlement or early survival of recruits by microhabitat, and the other one linking the activity of live barnacles with amelioration of the established ascidian recruits.

Barnacles, their empty tests or mimics thereof can either facilitate^36, 37^ or partially suppress^38^ the recruitment of solitary ascidians, depending on the particular species and habitat. Importantly, most small ascidians we found on live barnacles, both in the field and in the experiments, were either located inside an orifice or inside folds of the hosts’ test plates. Empty cockle shells (and smooth pebbles as well) do not provide any comparable shelter-like microhabitat. Consistently, shells in barnacle removal treatments, which demonstrated slightly higher ascidian recruitment rates than clear shells, were relatively rough with the remains of basal barnacle test plates. Similarly high ascidian recruitment rates on live barnacles and their empty tests suggest that the abundance of ascidians was rather affected by mechanical properties of barnacles than by their activity. This, in turn, is most likely caused by active larval choices leading to preferential settlement into folds, grooves and wrinkles, i.e. rugophilic behavior^39^ or selective elimination of openly settled recruits by predators.

There are no direct cues in favour of either process. Ascidian tadpoles, like many other invertebrate larvae, are considered rugophilic^37^. On the other hand, juvenile benthic invertebrates commonly undergo intense predation pressure, which is relaxed in refuges provided by structurally complex substrate. Older individuals are often less vulnerable to predators, and can safely outgrow their refuges^40^. Specifically, crevices on and between barnacles had been empirically proved to provide shelters increasing the survival of algal recruits otherwise grazed by limpets^41^. Although top-down control by consumers is generally thought to weaken with latitude^42^, predation is still important in community regulation at least in some sub-arctic and arctic marine benthic habitats^43–45^. Moreover, in absence of harsh environmental stress, facilitation is theoretically predicted to arise in response to consumer pressure^46^.

There a minor possibility that ascidian tadpoles selectively attach to barnacles based on some chemical cues. Also, the surfaces of primary substrates might accumulate a lot more silt than emerged barnacles and their empty tests, which could critically affect the survival of ascidian recruits^47^. Our unquantified observations, however, revealed no marked coverage of the exposed (at least for a year) cockle shells by soft sediment. In addition, barnacles, as filter feeders, can consume invertebrate planktonic larvae^48^ and were previously proved to suppress settlement in a bryozoan and several species of colonial ascidians. Consistent with our results, they had no negative effect on the recruitment of a solitary ascidian *Styela plicata* ^37^. Further direct experiments are necessary to reveal relative contributions of larval choices and selective juvenile mortality to the mechanism of facilitation.

After 11 months of exposure, ascidian recruits were larger on live barnacles than on empty tests. Given that more than 90% of these recruits were *Styela*-like, and the most locally common *Styela* species, *S*. *rustica*, has a single short annual spawning season in September^49^, live barnacles have seemingly increased their growth rate. The factors that could affect the small-scale variability in ascidian growth are poorly known. The adjacent sessile filter-feeding organisms are commonly expected to compete for food^50^, however the comparison of stable isotope ratios in tissues particularly between cirripeds and ascidians reveals no overlap in food sources^51^. Under certain hydrodynamic conditions barnacles can even increase feeding success of some neighbors, at least intraspecifically^52^. Live barnacles likely reduce the amount of silt in their immediate surroundings, but silt is only known to affect survival^47^ but not the growth rate in solitary ascidians. Possibly, the cirral activity of live barnacles modifies the flow so that the feeding success of ascidian recruits is improved.

According to annual growth ring counts in *Balanus crenatus*, the individual epibenthic patches on shells and pebbles in the shallow subtidal of the White Sea can persist at least for ten years^29^. Most patches are likely to start as an empty shell or a dropstone and further undergo a sequence of successive stages with barnacles as primary colonisers. Consistent with our present findings, almost no ascidians exist within these patches during the first four years of succession, and only on the fifth year, when barnacles already dominate, few can be found^28^. *Balanus crenatus* tests are smooth in young individuals and become folded as they grow. The supposed reliance of the facilitation on refuges provided to ascidian recruits predicts that barnacles have to grow old enough before their clusters get sufficiently packed with crevices and folds so that they can induce colonisation by ascidians. Later on, following the possible decline of barnacles caused by ageing, predation or overgrowth^10, 29, 45^, solitary ascidians likely stay on the empty tests left of them, which explains the observed accumulation of their biomass in this microhabitat.

Since solitary ascidians are evidently facilitated by barnacles and support a suit of dependent species themselves, they clearly act as a third-level foundation species in the facilitation cascade. Moreover, in the field solitary ascidians and nests of discord mussels (which were predominantly found on ascidians) provided substrate for most foliose red algae, which, in turn, have a diverse associated epibiota. This suggests that the facilitation cascade studied may actually comprise four or even five nested levels of foundation species bound by space-mediated positive interactions. The number of hierarchical facilitation levels in a community with multiple foundation species should be a critically important predictor of its stability and resilience. Indeed, the loss of a single foundation species can pull the ecosystem out of its stable state, causing dramatic changes to the local environment, abundance and diversity of dependent organisms, and nutrient fluxes^7^. The decline of foundation species caused by human activities broadly impacts the biota, and is therefore identified as an important global problem^53, 54^. When a community is shaped by several functionally distinct foundation species, the difference in their nested assemblages increases the total species diversity^10, 55^, so that a drop of any should reduce one. Foundation species themselves may exhibit interactions of variable strength and sign^11^ which makes them either self-sufficient (when they compete or do not interfere) or dependent (when some are facilitated by others). The first pattern with multiple foundation species on the same level of facilitative hierarchy may bolster the overall stability of the system similarly to the increased horizontal trophic heterogeneity *sensu* Persson^56^ through the generation of asynchronous dynamics in alternative resource provision channels^57^. Conversely, the latter pattern puts the dependent foundation species together with their nested assemblages to the risk of any stress underwent by the facilitators they depend on. The number of hierarchical levels in a facilitation cascade should consequently determine the degree to which the community-wide effects of the events involving a high-order facilitator are amplified. It is likely that the closer to the basal level is a facilitator engaged in a facilitation cascade, the more one’s decline jeopardizes the community. As a result, out of the communities with equal ‘horizontal facilitative heterogeneity’, those with more facilitation levels are potentially less stable, at least against disturbances affecting their primary facilitators.

Further developing the analogy between facilitation and trophic cascades, we should point out that some foundation species possibly share more than one cascade level. Here, for instance, red algae were markedly frequent on barnacles, discord mussel nests, and ascidians (Fig. 1). Alike the omnivory across trophic levels, i.e. ‘vertical trophic heterogeneity’^56^, the similar flexibility in resource allocation by a principal facilitator could also add to system stability.

Natural variation in the number of nested levels in facilitation cascades remains to be explored further. To date, both multi-level cascades examined are intertidal^19^ or subtidal (covered in this paper) marine epibenthic systems. Terrestrial facilitation cascades, however, possibly hide more nested levels than currently assumed. For instance, within the cascade recently found in a tree-epiphyte system, the epiphytic Spanish moss is facilitated by live oak *Quercus virginiana*, and, in turn, bolsters the abundance and diversity of the invertebrates both on oak limbs^21^ and underlying ground^22^. Similar hierarchical facilitation apparently drives many more plant-epiphyte systems. At the same time, various oak species from different habitats (with no evidence yet particularly for *Q*. *virginiana*) are dependent in their recruitment on nursing by shrubs, which provide shading critical for oak seedling survival^58, 59^. As a result, the multi-level ‘shrub-tree-epiphyte’ cascades may turn out to be quite common for terrestrial habitats.

In this study we have documented the facilitation cascade with at least three levels of nested foundation species, each hosting a diverse assemblage of dependent taxa. These findings add to the growing evidence^19^ that the webs of positive and negative interactions are much more alike in their complexity than it has been previously assumed. Foundation species may develop partially independent resource pathways sharing a cascade level (like alga and oyster in mangrove forests^14^ or ascidians and red algae here) and some probably operate at more than one level (like red algae here), potentially improving the overall system stability. We suggest that the cascades with multiple hierarchical facilitation levels and generally more complex positive interaction networks than known to date commonly occur in natural ecosystems, and anticipate further studies to link the properties of such networks to mechanisms of community assembly and functioning. Identification of the number of levels in a facilitation cascade, the position of each foundation species in a hierarchy and their functional interchangeability should be critically important to predict the trajectory of community change, and develop an adequate conservation or restoration policy.

## Methods

### Study area and field sampling

To review the field patterns of substrate surface utilisation by the foundation and dependent species we used the samples obtained by SCUBA divers at three subtidal locations in the vicinity of Solovetsky Islands (the Onega Bay of the White Sea) in July 2001–2014. Site 1 (12 m deep, 65°01.180′N, 35°39.721′E) was continuously sampled every year (28 samples in total), while Site 2 (15 m deep, 65°00.650′N, 35°41.650′E) was studied in 2003–2004 (6 samples), and Site 3 (12 m deep, 65°01.117′N, 35°40.039′E) in 2012 (2 samples) (Supplementary Table S4). A sample contained all the hard substrates (‘epibenthic patches’) visible on the sediment surface collected from a 1.00–1.44 m^2^ square frame haphazardly placed on the bottom. For each of these primary substrates we determined its surface area (see below), weighed red algae, and identified, counted and individually measured barnacles and ascidians. In most samples we also recorded the type of substrate they were attached to, and counted and identified (generally to species, except for sponges) any other sessile macrobenthic organisms larger than 0.3 mm by substrate type (details of the parameters recorded for each sample are summarised in Supplementary Table S4). Since 2011 we have also recorded the position of individual small ascidians (weighing 0.001 g and less) found on live barnacles in terms of ‘inside an orifice’ (either on mobile plates or inner sides of immobile plates), ‘outer inside folds’ (hidden inside the folds of the outer surface of immobile plates) and ‘outer open’ (openly attached to the outer surface of immobile plates). For each combination of sample and substrate type we calculated the Shannon diversity index (*H*’).

To assess the pattern of space provision we estimated the surface area of all the hard and flexible substrates potentially suitable for sessile organisms to attach. These were approximated from aperture length for barnacles and their empty tests, largest linear dimension for ascidians and discord mussels (*Musculus discors*), and weight for foliose red algae. Supplementary Methods describe the surface area estimation schemes for different substrates. Due to logistic constraints not all the proxies were available for each of the samples (see Supplementary Table S4). Surface areas of the secondary space providers were calculated for each sample and substrate type that the particular space provider occupied, so that we could compare the relative contributions to the substrate pool of the space providers from different microhabitats (e.g. barnacles living on primary substrates vs barnacles living on conspecifics).

To trace interspecific interactions between barnacles and solitary ascidians we assessed field variation in the biomass and juvenile density of the latter by substrate type. Specifically, we compared the densities of the smallest (weighing 0.001 g and less) ascidians between the primary substrate and live barnacles within each site using Wilcoxon matched-pairs signed-ranks test to reveal possible correlation between ascidian recruitment and the presence of barnacles. The mean values provided in the ‘Field sampling’ section of Results were calculated between the sites (n = 3) based on the data pooled by site, unless stated otherwise. Means are given ± S.E.

### Field experiments

To test the effect of barnacles on ascidian recruitment, we examined initially defaunated empty cockle shells, cockle shells with barnacles removed, cockle shells with empty barnacle tests, and cockle shells with live barnacles after 2 and 11 months of exposure. We collected empty Serripes groenlandicus shells, 4–7 cm long, in total 28 clear valves without barnacles (‘CS’ treatments) and 80 valves with 10–30 adult (4 or more growth bands, aperture length >5 mm) Balanus crenatus individuals on a convex side around the sites 1 and 3 in July 2010, 2011, and 2012. Each substrate was manually cleared of any macrobenthic organisms visible under a binocular microscope, both mobile and sessile, except live adult barnacles. Live barnacles were later removed from 14 random shells (‘BR’ treatments) and on 30 random shells they were transformed into empty tests by removing soft tissues and mobile shell plates (‘ET’ treatments), leaving alone with live barnacles the other 36 shells (‘LB’ treatments). Since barnacles technically could not be safely removed from fragile cockle shells completely, in contrast to CS treatments with smooth cockle shell surfaces, in BR treatments these were partially covered by fused basal plates of barnacle tests. We attached CS, BR, ET and LB shells convex side up in alternating sequences (5–7 cm from each other) to plastic grids anchored to the bottom at Site 1, and exposed them for 2 months (‘Short trials’) starting in early August 2010 and 2011, and for 11 months (‘Long trials’) starting in early August 2010, 2011 and 2012. The number of replicate treatments in each trial is summarised in Supplementary Table S5. In 2010 and 2011 we used 2 replicate CS treatments per 3 ET and 3 LB ones to maintain the proportion of primary substrate vs live barnacle surface areas roughly close to their proportion observed in the field. In 2012 we did not use ET and doubled the number of CS to get more ascidans from cockle shells for individual measurements. BR treatments were only used in 2011 and 2012. At the end of the trials we collected the shells, and examined their upper (convex) sides in the laboratory as separate samples, counting and individually measuring barnacles, their empty tests and solitary ascidians by substrate type, and visually estimating barnacle covers and lateral contiguity percentage for each barnacle or empty test. The following substrate types were recognised: (i) cockle shell in CS; (ii) cockle shell with barnacle traces in BR; (iii) cockle shell and (iv) empty barnacle tests in ET; (v) cockle shell, (vi) outer surface of live barnacles and (vii) any location inside the opercular orifice of a live barnacle, including the inner surface of immobile shell plates and the surface of mobile plates (hereafter ‘inside an orifice’). The areas were separately approximated for each of these substrates from the linear measurements of the shells and tests (see Supplementary Methods). The outer areas of barnacles and empty tests were corrected for their lateral contiguity, and the cockle shell surface areas in LB and ET were corrected for adult barnacle (and empty test) covers. Throughout the experiments some barnacles recruited to CS and ET, but there were no ascidians on their surface. Also, a few of the adult barnacles in LB died by the end of the trials; the ascidians found on them (16 individuals or 3% of the total 539 ascidians recorded in LB) were ignored in further analysis. By the end of the short trials most ascidians were too small for accurate identification even to the genus level.

The recruitment rates of ascidians on cockle shells, barnacles and their empty tests in the short and long trials 2010–2011 and 2011–2012 were compared between the experimental units with type III sum of squares 2-way ANOVA. The factors were Treatment (fixed with 3 or 4 levels: CS, BR (2011–2012 only), ET, LB), Exposure (fixed with 2 levels: 2 and 11 months), and the interaction thereof, and the response variable was the number of solitary ascidians per area of the corresponding substrate. Because of the absence of BR in 2010–2011, the two years were analysed separately. For these analyses we only used the abundances of ascidians attached to the principal substrate for each experimental unit, i.e. shell surface in CS and BR, total (inner and outer pooled) empty tests’ surface in ET and total (inner and outer pooled) live barnacles’ surface in LB. The recruitment rates of barnacles on cockle shells, conspecifics and their empty tests in 2010–2011 and 2011–2012 were analysed in a similar way. In 2012–2013 the abundances of barnacle recruits were compared between LB and CS using the Mann-Whitney U-test. Since the 2012–2013 experiment had no short trial and ET, but both 2011–2012 and 2012–2013 had BR, we also examined the abundance of ascidians by the 2-way type III sum of squares ANOVA with factors Treatment (fixed with 3 levels: CS, BR, LB) and Year (fixed with 2 levels: 2011–2012 and 2012–2013). Here and below, Year was considered a fixed effect since it had insufficient number of levels to assess one as random.

As the immediate presence of adult barnacles could affect the recruitment of ascidians to cockle shell surfaces, we performed a separate 2-way type III sum of squares ANOVA with ascidian densities on bare cockle shells from CS and cockle shells from LB exposed for 11 months as a response variable. The factors were Treatment (fixed with 2 levels: bare cockle shells and cockle shells with live barnacles), Year (fixed with 3 levels: 2010–2011, 2011–2012 and 2012–2013) and their interaction. To assess the preferences of ascidians for different microhabitats within LB we performed pairwise comparisons of ascidian densities outside and inside the orifice of live barnacles by Wilcoxon matched-pairs signed-ranks tests across the three years of manipulations and within each year. Treatments with different exposures were analysed separately.

To examine the effect of barnacles on growth rate of ascidian recruits we compared individual weights of ascidians between substrates by one-way ANOVA with the factor Treatment in 2011–2012, when ascidians were found in CS, ET and LB, and Mann-Whitney U-tests in 2010–2011 and 2012–2013 when there were no ascidians in CS or there were no ET treatments, respectively. The response variable was the individual weight of ascidians after 11 months of exposure averaged by experimental unit.

Homogeneity of variances was checked using Cochran’s test, and the data was square root transformed where needed to achieve it. Following Quinn and Keough^60^, we regarded variance homogeneity the critical condition for usage of ANOVA, also taking into account that the power of the analysis could be reduced by left-skewness of the data^61^. Each ANOVA was followed by Tukey HSD tests for pairwise comparisons of the means. The proportions of *Molgula*-like and *Styela*-like ascidian recruits were compared between LB and ET using 2 × 2 contingency table Chi-square test. Unless otherwise specified, the significance level was 0.05 for all the tests.

The data that support the findings of this study are available from the corresponding author upon reasonable request.

## Electronic supplementary material

Supplementary Information
