# Supplementary material for: Cockles, barnacles and ascidians compose a subtidal facilitation cascade with multiple hierarchical levels of foundation species
Source: Sci Rep. 2017 Mar 22;7:237. doi: 10.1038/s41598-017-00260-2 (PMC5427999; doi:10.1038/s41598-017-00260-2)
Supplement: Supplementary file 1 — Supplementary Information [file 41598_2017_260_MOESM1_ESM.pdf]

Cockles, barnacles and ascidians compose a subtidal facilitation cascade with multiple hierarchical levels of foundation species

E. Yakovis, A. Artemieva

Supplementary Information

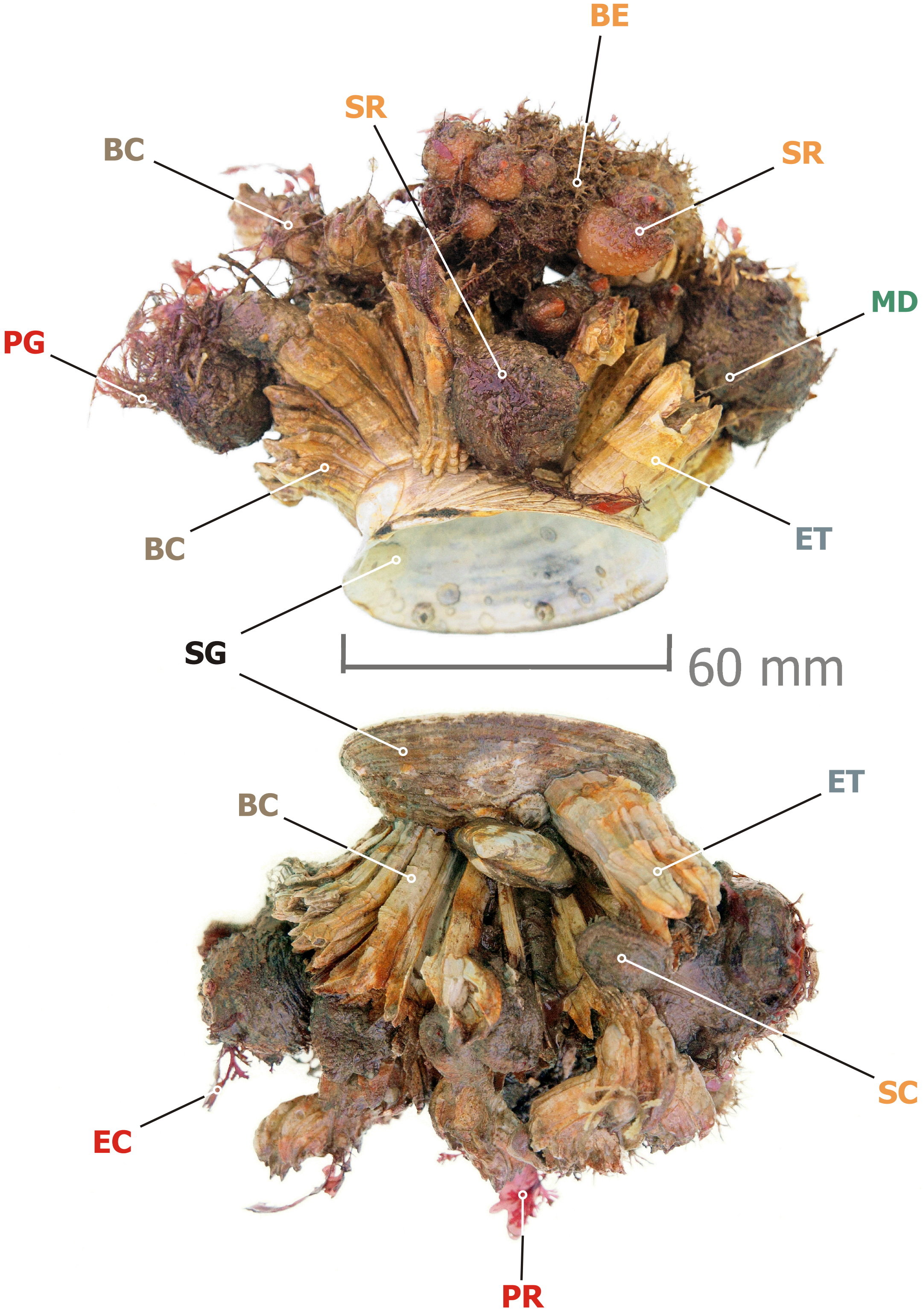

Supplementary Figure S1. A typical epibenthic patch on a cockle shell.
Sampled at Site 1 on July 29, 2014. SG – an empty *Serripes groenlandicus* shell, BC – barnacles *Balanus crenatus*, ET – empty barnacle tests, MD – a dicord mussel *Musculus discors*, embedded into ascidian tunic; solitary ascidians: SR – *Styela rustica*, SC – *Styela coriacea*, BE – *Boltenia echinata*; red algae: PR – *Phycodrys rubens*, PP – *Ptilota gunneri*, EC – *Euthora cristata*.

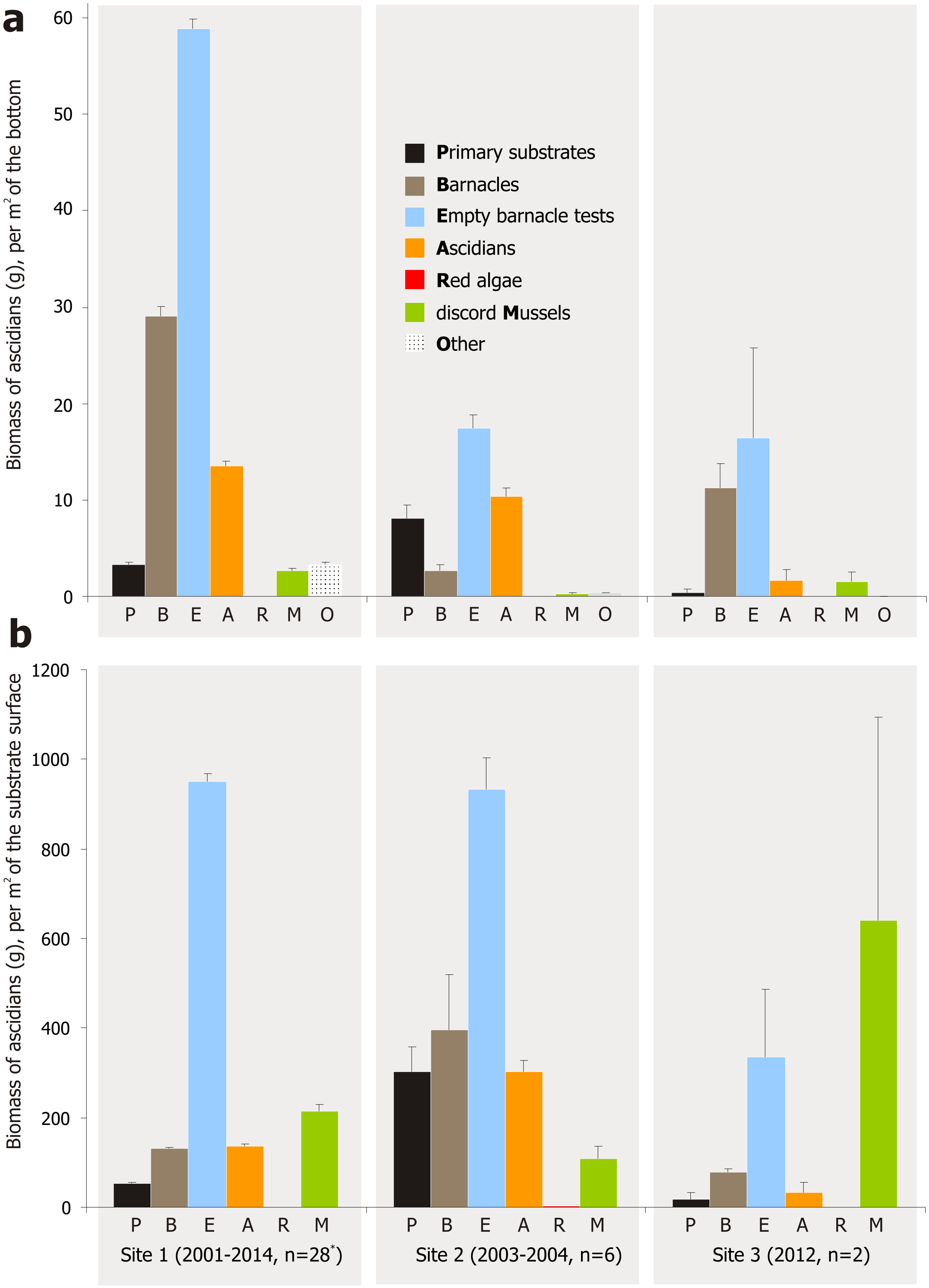

Supplementary Figure S2. Average biomass of solitary ascidians in the field by site and substrate type. Biomass of solitary ascidians per m^2 of the bottom (**a**). Biomass of solitary ascidians per m^2 of the approximated substrate surface area (**b**). * – the biomass of ascidians on discord mussels at Site 1 was only available in 2001–2004 and 2007–2014 (n=24).

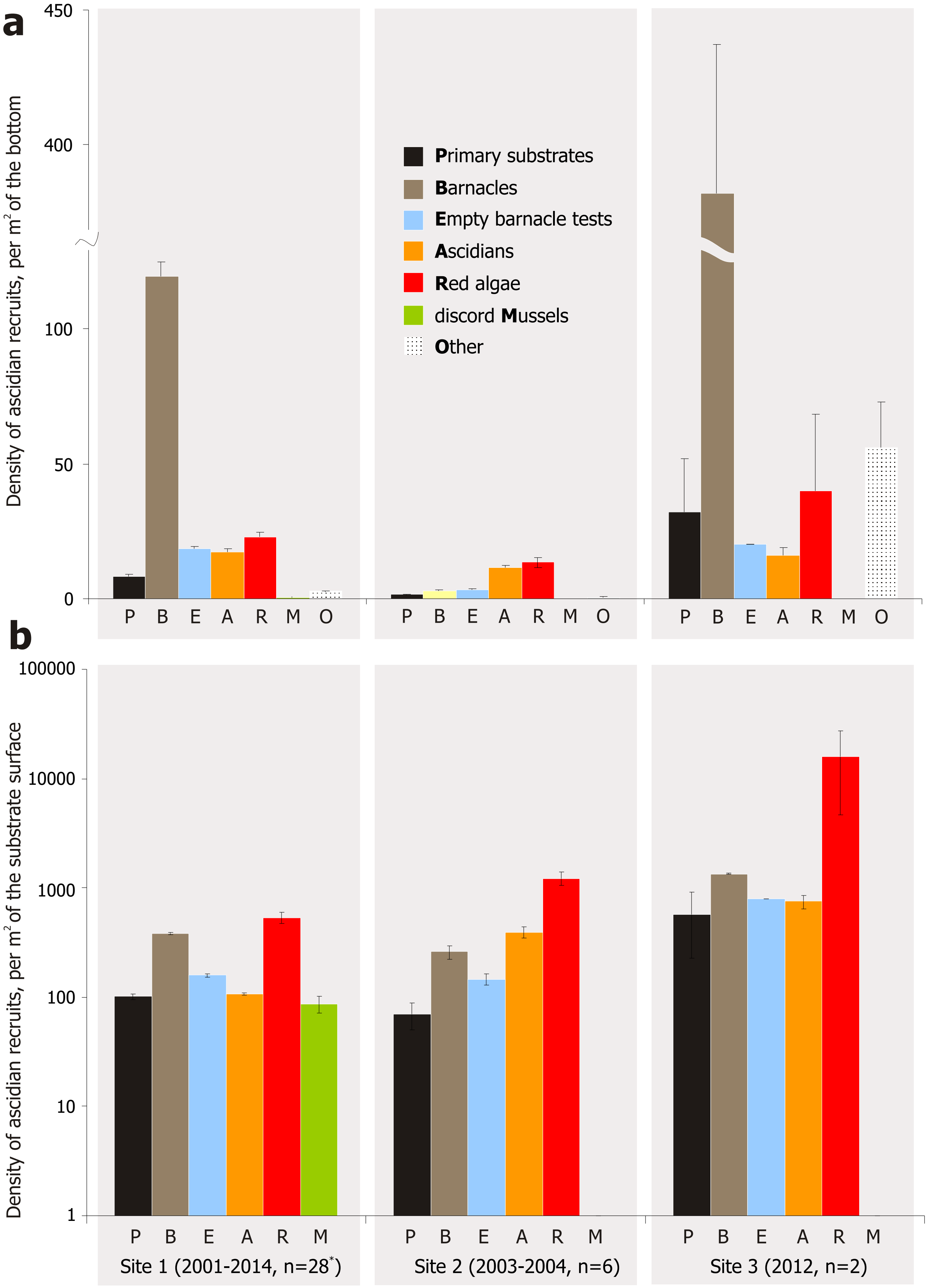

Supplementary Figure S3. Recruitment of solitary ascidians in the field by site and substrate type. Mean numbers of ascidian recruits (individual weight 0.001 g and less) per m^2 of the bottom (**a**). Mean numbers of ascidian recruits per m^2 of the approximated substrate surface area (**b**). * – the abundances of ascidian recruits on red algae at Site 1 were available only in 2001–2004 and 2011–2014 (n=16); the abundances of ascidian recruits on dicord mussels at Site 1 were available only in 2001–2004 and 2007–2014 (n=24).

Supplementary Table S1. Field substrate manipulation experiment: the results of ANOVA on abundances of solitary ascidians found on the surface of initially clear *Serripes groenlandicus* shells with (LB) and without (CS) live barnacles exposed for 11 months in 2010–2013. Square root transformed densities per m^2 of *Serripes* shell surface used as a response variable. Variances were homogeneous after the transformation. The results of Tukey HSD post-hoc test for Treatment levels were nonsignificant (p=0.976). CS – empty *Serripes groenlandicus* shells. LB – *Serripes groenlandicus* shells with live *Balanus crenatus*.

Source of variation	df	SS	F	p	Mean±S.E. by Treatment level (m^{-2})	
					CS	LB
Treatment (fixed)	1	13	0.179	0.674	94±30	141±56
Year (fixed)	2	958	6.609	**0.003**		
Treatment x Year (fixed)	2	172	1.186	0.317		
Error	38	2754				

Supplementary Table S2. Field substrate manipulation experiment: the results of ANOVA on individual mean weights of solitary ascidians found on initially clear empty *Serripes groenlandicus* shells, empty *Balanus crenatus* tests and live *Balanus crenatus* exposed for 11 months in 2011–2012. Square root transformed mean individual weight of ascidians in an experimental unit used as a response variable. Variances were homogeneous after the transformation. Ascidians found on *Serripes* shells in ET and LB treatments and on empty barnacle tests in LB treatments were ignored. There were no ascidians on live barnacles in ET treatments. The results of Tukey HSD post-hoc tests are indicated by letters 'a' and 'b' following the means. Significantly (p<0.001) different means have no letter in common. CS – empty *Serripes groenlandicus* shells. ET – *Serripes groenlandicus* shells with empty *Balanus crenatus* tests. LB – *Serripes groenlandicus* shells with live *Balanus crenatus*.

Source of variation	df	SS	F	p	Mean±S.E. by Treatment level (g)		
					CS	ET	LB
Treatment (fixed)	2	0.008	67.597	0.000	0.0010±0.0000 a	0.0012±0.0001 a	0.0066±0.0008 b
Error	13	0.001					

Supplementary Table S3. Field substrate manipulation experiment: the results of ANOVA on abundances of barnacle recruits found on initially clear empty *Serripes groenlandicus* shells, empty *Balanus crenatus* tests and live *Balanus crenatus* exposed for 2 and 11 months in 2010–2012. Densities per 1 m^2 of substrate surface used as a response variable. Barnacles found on *Serripes* shells in ET and LB treatments and on empty barnacle tests in LB treatments were ignored. The results of Tukey HSD post-hoc tests are indicated by letters 'a' and 'b' following the means. Significantly ($p<0.05$) different means have no letter in common. CS – empty *Serripes groenlandicus* shells. ET – *Serripes groenlandicus* shells with empty *Balanus crenatus* tests. LB – *Serripes groenlandicus* shells with live *Balanus crenatus*.

Source of variation	df	SS	F	p	Mean±S.E. by Treatment level (m^{-2})		
					CS	ET	LB
2010-2011 (variances homogeneous, no transformation needed)							
Treatment (fixed)	2	2501369	4.640	**0.015**	137±95 a	405±114 ab	761±147 b
Exposure (fixed)	1	469724	1.743	0.194			
Treatment x Exposure (fixed)	2	17774	0.033	0.968			
Error	42	11320251					
2011-2012 (variances heterogeneous despite the transformations)							
Treatment (fixed)	2	2139404	0.703	0.504	960±702 a	845±190 a	1417±340 a
Exposure (fixed)	1	5857659	3.850	0.061			
Treatment x Exposure (fixed)	2	3729512	1.223	0.310			
Error	26	39561834					

Supplementary Table S4. Field sampling details. In our previous communtications[10,45] the Sites 1, 2 and 3 were referred to as 'Site 1'[10,45], 'Site 2'[10] and 'Site 2'[45], respectively. Since 2011 the position was recorded for the ascidian recruits found on live barnacles, i.e. 'inside an orifice', 'on an outer walls openly', and 'on an outer walls inside a fold' (see Methods for details).

Sample ID	Site	Sampling date	Area sampled, m^2	All sessile organisms counted by substrate	Ascidian recruits (weighing 1 mg and less) counted by substrate	Barnacles and their empty tests measured	Solitary ascidians weighed by substrate	Red algae weighted	Discord mussels measured
A-IX	1	09.07.01	1.44	+	+	only totally	+	only totally	only totally
A-X	1	17.07.01	1.44	+	+	only totally	+	only totally	only totally
A-XI	1	24.07.01	1.44	+	+	only totally	+	only totally	only totally
A-XII	1	09.07.02	1.44	+	+	only totally	+	only totally	only totally
A-XIII	1	15.07.02	1.44	+	+	only totally	+	only totally	only totally
A-XIV	1	15.07.02	1.44	+	+	only totally	+	only totally	only totally
D-I	2	14.07.03	1.44	+	+	only totally	+	by substrate	only totally
A-XV	1	19.07.03	1.44	+	+	by substrate	+	only totally	only totally
D-II	2	23.07.03	1.44	+	+	by substrate	+	only totally	only totally
A-XVI	1	25.07.03	1.44	+	+	by substrate	+	only totally	only totally
D-III	2	07.07.04	1.44	+	+	by substrate	+	only totally	only totally
D-IV	2	07.07.04	1.44	+	+	by substrate	+	only totally	only totally
A-XVII	1	10.07.04	1.00	+	+	by substrate	+	only totally	only totally
D-V	2	15.07.04	1.44	+	+	by substrate	+	only totally	only totally

D-VI	2	15.07.04	1.44	+	+	by substrate	+	only totally	only totally
A-XVIII	1	18.07.04	1.00	+	+	by substrate	+	only totally	only totally
A-sXIX	1	17.07.05	1.00	-	except on red algae	by substrate	+	only totally	-
A-sXX	1	17.07.05	1.00	-	except on red algae	by substrate	+	only totally	-
A-sXXI	1	24.07.06	1.00	-	except on red algae	by substrate	+	only totally	-
A-sXXII	1	24.07.06	1.00	-	except on red algae	by substrate	+	only totally	-
A-sXXIII	1	12.07.07	1.00	-	except on red algae	by substrate	+	by substrate	by substrate
A-sXXIV	1	17.07.07	1.00	-	except on red algae	by substrate	+	by substrate	by substrate
A-sXXV	1	04.07.08	1.00	-	except on red algae	by substrate	+	by substrate	by substrate
A-sXXVI	1	21.07.08	1.00	-	except on red algae	by substrate	+	by substrate	by substrate
A-sXXVII	1	13.07.09	1.00	-	except on red algae	by substrate	+	by substrate	by substrate
A-sXXVIII	1	28.07.09	1.00	-	except on red algae	by substrate	+	by substrate	by substrate
A-sXXIX	1	14.07.10	1.00	-	except on red algae	by substrate	+	by substrate	by substrate
A-sXXX	1	21.07.10	1.00	-	except on red algae	by substrate	+	by substrate	by substrate
A-sXXXI	1	24.07.11	1.00	within 12 random patches	+	by substrate	+	by substrate	by substrate
A-sXXXIII	1	28.07.12	0.25+0.75	within 0.25 m^2	within 0.25 m^2	by substrate	+	by substrate	by substrate
I-sI	3	03.08.12	0.25+0.75	within 0.25 m^2	within 0.25 m^2	by substrate	+	by substrate	by substrate
I-sII	3	03.08.12	0.25+0.75	within 0.25 m^2	within 0.25 m^2	by substrate	+	by substrate	by substrate
A-sXXXV	1	29.07.13	0.25+0.75	within 0.25 m^2	within 0.25 m^2	by substrate	+	by substrate	by substrate
A-sXXXVI	1	03.08.13	0.25+0.75	within 0.25 m^2	within 0.25 m^2	by substrate	+	by substrate	by substrate
A-sXXXVII	1	29.07.14	0.25+0.75	within 0.25 m^2	within 0.25 m^2	by substrate	+	by substrate	by substrate
A-sXXXVIII	1	02.08.14	0.25+0.75	within 0.25 m^2	within 0.25 m^2	by substrate	+	by substrate	by substrate

Supplementary Table S5. Experimental design: the numbers of experimental units used in the field experiments by treatment and trial. BR – *Serripes groenlandicus* shells with *Balanus crenatus* removed. CS – empty *Serripes groenlandicus* shells. ET – *Serripes groenlandicus* shells with empty *Balanus crenatus* tests. LB – *Serripes groenlandicus* shells with live *Balanus crenatus*.

Year	Started on	Short trial (2 months)					Long trial (11 months)				
		Finished on	Treatment				Finished on	Treatment			
			BR	CS	ET	LB		BR	CS	ET	LB
2010-2011	August 5-7, 2010	October 3, 2010		4	6	6	July 7-17, 2011		8	12	12
2011-2012	August 5-7, 2011	October 2, 2011	4	4	6	6	July 7-15, 2012	4	4	6	6
2012-2013	August 10, 2012	-					July 6-12, 2013	6	8		6

Supplementary Methods. Substrate surface area estimation procedures.

Primary substates. We measured the length (L) of *Serripes* shells (which were the most frequent primary substrate) and used it as a proxy for surface area estimation (S) according to the previously estabilished relationship[10]:

$$S = 0.8L^2+15.91L-284.48.$$

Surface areas of the *Serripes* shells with numerous traces of barnacles, other shells, gravel and pebbles were measured directly accurate to 0.5 cm^2 either using the aluminum foil wrapping method or by applying a transparent grid. Most primary substrates had a distinct visual border of the part that was submerged into the soft sediment at the time of sampling, in which case we considered only the area that was above the sediment surface.

Live barnacles. Surface areas of live barnacles were calculated by adding up their outer (S_{out}) and inner (S_{in}, i.e. *tergum* + *scutum* + inner parts of immobile plates) surface areas both approximated from the aperture length (L) according to the previously estabilished relationships[10]:

$$S_{out} = 5.5L^{2.1215} \text{ and } S_{in} = 0.64L^2+0.37L.$$

Empty barnacle tests. We considered the sum of the above relationships also suitable to estimate surface areas of empty barnacle tests. In fact, according to our visual field observations, empty barnacle tests at the sites studied are 70-90% filled with soft sediment, which screens most of their inner surface from being occupied by epibionts. Consistently, sessile organisms found inside an intact empty test as a rule only occured at its distal one third part, close to the orifice. We thus assumed that the surface area provided by an empty barnacle test available for epibionts is approximately similar to the one provided by a same size live barnacle.

Solitary ascidians. Based on their morphology, the surface areas for *Styela coriacea*, *Dendrodoa grossularia* and juvenile *Styelidae* were approximated by hemispheres with the diameter equal to the largest linear dimension of an ascidian individual. Similarly, for surface area estimation purposes *Molgula spp.* and *Boltenia echinata* were modeled by spheres (the diameter equal to their largest linear dimension), while *Styela rustica* were modeled by cylinders without a bottom, which had the height and diameter both equal to the largest linear dimension of an ascidian individual.

Foliouse red algae. Surface areas (S, in mm^2) of foliouse red algae were roughly estimated from their wet weight (W, in grams) according to the relationship based on the random sample of *Phycodrys rubens* which dominated by biomass at all the sites studied:

$$S = 8907.5W, R^2 = 0.4677, \text{n} = 198.$$

The surface areas of algal blades used to develop the above relationship were determined by laying on graph paper with 1 mm grid.

Discord mussels. Since the space provided by discord mussels was actually the surface of their bissus-made nests, most of which were half-embeded in ascidian tunics or clustered with each other, we approximated the surface area they contributed to the habitat as hemispheres with diameters equal to the lengths of the bivalves
